# AI-SCoRE (artificial intelligence-SARS CoV2 risk evaluation): a fast, objective and fully automated platform to predict the outcome in COVID-19 patients

**DOI:** 10.1007/s11547-022-01518-0

**Published:** 2022-08-29

**Authors:** Anna Palmisano, Davide Vignale, Edda Boccia, Alessandro Nonis, Chiara Gnasso, Riccardo Leone, Marco Montagna, Valeria Nicoletti, Antonello Giuseppe Bianchi, Stefano Brusamolino, Andrea Dorizza, Marco Moraschini, Rahul Veettil, Alberto Cereda, Marco Toselli, Francesco Giannini, Marco Loffi, Gianluigi Patelli, Alberto Monello, Gianmarco Iannopollo, Davide Ippolito, Elisabetta Maria Mancini, Gianluca Pontone, Luigi Vignali, Elisa Scarnecchia, Mario Iannacone, Lucio Baffoni, Massimiliano Sperandio, Caterina Chiara de Carlini, Sandro Sironi, Claudio Rapezzi, Luca Antiga, Veronica Jagher, Clelia Di Serio, Cesare Furlanello, Carlo Tacchetti, Antonio Esposito

**Affiliations:** 1 Experimental Imaging Center, IRCCS San Raffaele Scientific Institute, Via Olgettina 60, Milan, Italy; 2 School of Medicine, Vita-Salute San Raffaele University, Via Olgettina 58, Milan, Italy; 3 Centro Universitario Di Statistica Per Le Scienze Biomediche, Vita-Salute San Raffaele University, Milan, Italy; 4 Porini Srl, Milan, Italy; 5 Orobix Life Srl, Bergamo-Rovereto, Italy; 6 GVM Care & Research Maria Cecilia Hospital, Cotignola, Italy; 7 Ospedale Di Cremona, Cremona, Italy; 8 ASST Bergamo Est - Bolognini Hospital, Seriate, Italy; 9 Guglielmo da Saliceto Hospital, Piacenza, Italy; 10 Ospedale Maggiore, Bologna, Italy; 11 San Gerardo Hospital, Monza, Italy; 12 Centro Cardiologico Monzino IRCCS, Milan, Italy; 13 Parma University Hospital, Parma, Italy; 14 ASST Valtellina and Alto Lario, Eugenio Morelli Hospital, Sondalo, Italy; 15 San Giovanni Bosco Hospital, ASL Città di Torino, Turin, Italy; 16 Casa di Cura Villa dei Pini, Civitanova Marche, Italy; 17 ICC Istituto Clinico Casalpalocco, Rome, Italy; 18 San L. Mandic Hospital, Merate, Italy; 19 ASST Papa Giovanni XXIII, Bergamo, Italy; 20 Cardiologic Centre, University of Ferrara, Ferrara, Italy; 21 Microsoft Corporation, Milan, Italy

**Keywords:** Artificial intelligence, COVID-19, Computed tomography, Calcium score

## Abstract

**Purpose:**

To develop and validate an effective and user-friendly AI platform based on a few unbiased clinical variables integrated with advanced CT automatic analysis for COVID-19 patients’ risk stratification.

**Material and Methods:**

In total, 1575 consecutive COVID-19 adults admitted to 16 hospitals during wave 1 (February 16-April 29, 2020), submitted to chest CT within 72 h from admission, were retrospectively enrolled.

In total, 107 variables were initially collected; 64 extracted from CT. The outcome was survival.

A rigorous AI model selection framework was adopted for models selection and automatic CT data extraction. Model performances were compared in terms of AUC. A web–mobile interface was developed using Microsoft PowerApps environment. The platform was externally validated on 213 COVID-19 adults prospectively enrolled during wave 2 (October 14-December 31, 2020).

**Results:**

The final cohort included 1125 patients (292 non-survivors, 26%) and 24 variables. Logistic showed the best performance on the complete set of variables (AUC = 0.839 ± 0.009) as in models including a limited set of 13 and 5 variables (AUC = 0.840 ± 0.0093 and AUC = 0.834 ± 0.007). For non-inferior performance, the 5 variables model (age, sex, saturation, well-aerated lung parenchyma and cardiothoracic vascular calcium) was selected as the final model and the extraction of CT-derived parameters was fully automatized. The fully automatic model showed AUC = 0.842 (95% CI: 0.816–0.867) on wave 1 and was used to build a 0–100 scale risk score (AI-SCoRE). The predictive performance was confirmed on wave 2 (AUC 0.808; 95% CI: 0.7402–0.8766).

**Conclusions:**

AI-SCoRE is an effective and reliable platform for automatic risk stratification of COVID-19 patients based on a few unbiased clinical data and CT automatic analysis.

**Supplementary Information:**

The online version contains supplementary material available at 10.1007/s11547-022-01518-0.

## Introduction

Since early 2020, SARS-CoV-2 infection reached the level of pandemic, becoming a public health emergency of international concern [1]. SARS-CoV-2 infection is highly transmissible, and COVID-19 progression is often abrupt, rapidly precipitating from a mild symptomatic disease to respiratory failure requiring critical care [2].

To reduce the burden on high-intensity hospitals, a fast and reliable prediction method to sort patients at risk of severe illness and death at the admission to emergency department (ED), represents a crucial need for patient’s management and general public health aims.

Several features have been suggested as predictors of COVID-19 patients’ outcome; among them, age, sex, comorbidities, biomarkers of systemic inflammation [3] and a plethora of prognostic models [4, 5] including these variables have been proposed to improve patients risk stratification. However, the clinical applicability of these models is far from being achieved mainly due to methodological flaws or underlying biases [6]. In particular, limited sample size, lack of external validation, quality data concern (missing variable and imputation of data, unstandardized values, qualitative or semiquantitative measurements) and different definition of outcome have been limiting to various extent these proposed solutions [6]. Moreover, reliability and rapidity of the collection of these data is challenging and not always compatible with the stressful situation in overwhelmed hospitals facing a pandemic. In particular, anamnestic data, including referred number and type of comorbidities, may be influenced by the patient’s clinical condition, age and mental status, as well as by socioeconomic context. Moreover, laboratory tests may require long turnaround time, particularly in overwhelmed laboratories, and may have different reference values among laboratories and countries. Finally, radiological features are operator-dependent and time-consuming.

The integration of features automatically extracted by medical images within predictive algorithms based on artificial intelligence (AI), promises to address these issues, offering the possibility to derive prediction models based on measures that are completely independent from patient’s and operator’s subjectivity.

Chest CT has been widely adopted in clinical practice during COVID-19 pandemic, to support clinical decision making [7].

Chest CT allows to assess COVID-19-related pneumonia severity [8], identifying complications [9], and allows a comprehensive patients phenotyping, providing information about cardiovascular risk [10–14], pulmonary hypertension [15] and patients fragility [16]. Moreover, chest CT is suitable for AI driven differential diagnosis [17] and automatic extraction of relevant features [18], overcoming biases related to subjective evaluation and issues related to scarcity of specialized physicians during a pandemic.

The goal of this study is to design and validate an AI platform (AI-SCoRE, Artificial Intelligence – Sars Covid prognostic Risk Evaluation), with both computer and mobile phone interfaces, based on a reliable, unbiased and fast algorithm, able to automatically elaborate DICOM chest CT images and clinical data, collected from patients at the first appearance of COVID-19 symptoms and return a reliable prognostic risk score.

## Materials and methods

### Study design

This clinical study (AI-SCoRE; NCT04834934) included a retrospective series of 1575 consecutive COVID-19 adults, admitted to the emergency department (ED) of 16 hospitals in Northern Italy (detailed list in Supplementary Material) during the first wave of pandemic (February 16-April 29, 2020) and a cohort of 213 consecutive COVID-19 adults prospectively enrolled during the second wave of the pandemic (October 14-December 31, 2020) at IRCCS San Raffaele Hospital, Milan, Italy, for prospective external validation.

The local Ethic Review Board of each Institution approved the study.

Collected clinical and CT parameters are listed in Supplementary Material.

Data collection was concluded on June 30, 2020, for the first wave cohort and the February 28, 2021, for the second wave cohort.

**Fig. 1:**
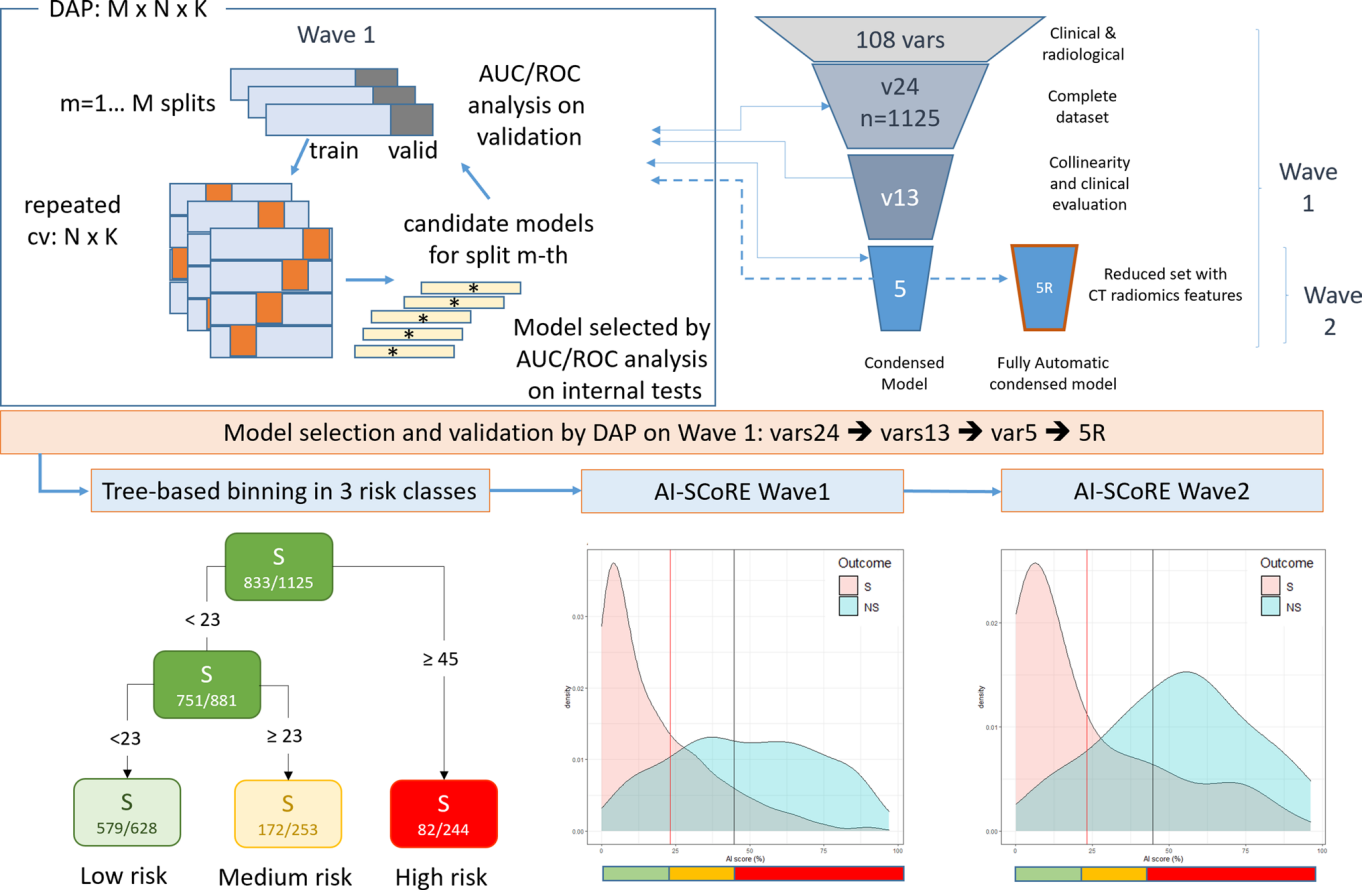
Workflow of the AI-SCoRE model selection procedure and validation. AI Score risk partition in three bins, defining the low-, medium- and high-risk groups and AI-SCoRE estimated class densities, with the indication of the two thresholds defining the bin partition, for wave 1 and wave 2

The study was developed through six steps: (1) creation of a training dataset including the more significant clinical and CT variables manually extracted from CT images and medical records in patients from first wave, to test different multivariate models for binary classification targets prediction models; (2) definition of a prediction model and progressive reduction of the number of variables included, in order to obtain a model with only essential, unbiased and automated clinical and CT variables, with non-inferior performance when compared to the models including wider variable sets; (3) automation of the extraction of the CT quantitative features; (4) selection of the final automatic model for building the “AI-SCoRE” and identification of the AI-SCoRE threshold values able to identify three different risk classes; (5) design and deployment of a user-friendly smartphone and PC interface to manage AI-SCoRE platform; and (6) external validation of AI-SCoRE on a prospective cohort belonging to the second wave (Fig. 1).

### AI model

#### Variables selection and model development

A three-layered Data Analysis Plan (DAP) extending the 10 × 5 repeated CV design of the FDA-led MAQC-II project [19] was adopted for model development and selection. The first wave data were split M = 10 times into training and test with *p* = 0.7 proportion (n_train = 789, n_test = 336). For each split, a NxK repeated cross-validation was applied to the training data in the split (N = 10, K = 5). Candidate models were developed applying functions of the caret framework [20] within the R statistical environment (R-0.4.03 version). As the primary model selection metric, we considered the area under the curve (AUC) for the receiver operating curve (ROC). The model set included nine different multivariate models typically used in AI for binary classification targets. All models were first trained on a group of 24 variables, progressively reduced to a set of 5 variables. The caret framework provided the automated collection of an optimal parameter tuning for each model type based on the internal repeated cross-validation scheme. For model selection, the average of AUC over the internal tests were collected for each split for a total of 50 models per split and then averaged over the splits. For model evaluation, mean AUC over the M test sets was considered.

### Automatic extraction of CT features

#### Well-aerated lung volume and pneumonia features

A combination of two pre-trained deep learning models was used to automatically extract quantitative pneumonia features from CT images. Lung masks were obtained using the publicly available R231 model [21], a 2D U-net operating on individual slices was trained on a dataset representative of different consolidated lung involvement (Vol R231). For comparison, a second pre-trained segmentation model based on the volumetric U-net or V-net [22] available as part of the NVIDIA Clara COVID-19 Collection [23] was employed for pneumonia extraction (pneumonia_C). In order to enrich the description of COVID-19 lesions beyond what extracted from the pneumonia model, the relative fractions of voxels within the lung mask corresponding to the HU intervals for GGO (-780, -570), semi-consolidation (-570, -290) and consolidation (≥ -290) proposed by Esposito et al. [8] were computed and used as additional features: well-aerated lung volume (WALV%_E), ground glass opacities (GGO%_E), semi-consolidations (SC%_E), consolidations and overall interstitial involvement (GGO-SC%_E) (details in Supplementary Methods).

#### Total cardiovascular thoracic calcium

Total cardiovascular thoracic calcium volume including coronary arteries, aortic valve and thoracic aorta calcium volumes was obtained using a multistep approach (details in Supplementary Methods). The automated estimator strongly correlates with the manual segmentation (R = 0.844 p = 2.2e-16). An analytical validation experiment with a robust regression model (PaBablok method) is reported in Fig. 1S.

An example of the automatic features extraction is provided in Figure S2.

## Results

### Training dataset

**Fig. 2:**
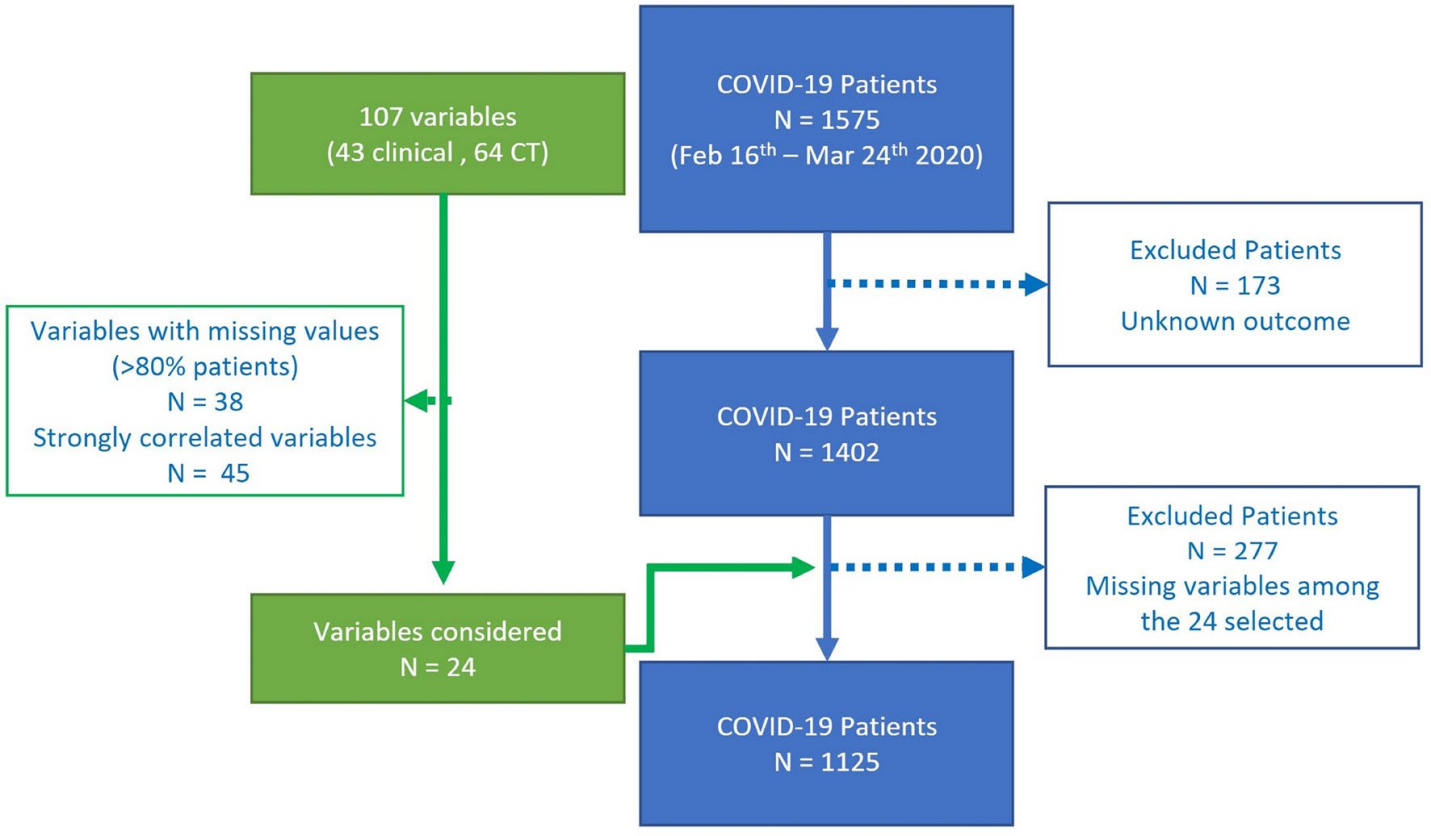
Enrollment flowchart and data cleaning process workflow

From a total of 1575 consecutive patients and 107 variables (43 different demographic, clinical and laboratory test variables and 64 variables from chest CT), according to data cleaning steps (Fig. 2), the final cohort included 1125 patients and 24 variables.

Patients’ main clinical and manually extracted CT features were reported in Tables 1, also accounting for patients’ outcome (NS = 292, 26% and S = 833; 74%).

**Table 1:** Clinical, demographic, laboratory and CT features of wave 1 population

	Overall (N = 1125)	Survivors (N = 833)	Non-Survivors (N = 292)	Adj. p-value
*Clinical characteristics*				
Male Sex, n (%)	763 (68.1%)	542 (65.1%)	221 (75.7%)	0.001
Age, y.o. (median [IQR])	69.5 [59, 77]	66 [57, 74]	77 [70, 83]	< 0.001
Oxygen saturation in ambient air, % (median [IQR])	92 [88, 95]	93 [90, 96]	89 [82, 93]	< 0.001
Hypertension, n (%)	643 (57.2%)	453 (54.4%)	190 (65.1%)	0.002
Diabetes, n (%)	217 (19.3%)	141 (16.9%)	76 (26.0%)	0.001
Chronic obstructive pulmonary disease, n (%)	115 (10.2%)	69 (8.3%)	46 ( 15.8%)	< 0.001
Known active neoplasia, n (%)	56 (5%)	37 (4.4%)	19 (6.5%)	0.162
Heart disease, n (%)	206 (18.3%)	112 (13.4%)	94 (32.2%)	< 0.001
*Laboratory tests*				
Hemoglobin (g/dl)	13.9 [12.5, 14.9]	14.0 [12.7, 14.9]	13.5 [12.0, 14.6]	< 0.001
White blood cells (mm^3^)	6760 [5000, 9490]	6540 [4900, 9220]	7260 [5480, 10568]	< 0.001
Creatinine (mg/dl)	1.01 [0.84, 1.28]	0.98 [0.81, 1.19]	1.21 [0.96, 1.76]	< 0.001
C Reactive Protein (mg/dl)	8.37 [3.18, 14.94]	6.97 [2.50, 13.01]	12.73 [6.87, 19.40]	< 0.001
*Coronary calcium and stent*				
Absent Present Stent	339 (30.1%) 800 (62.2%) 86 (7.6%)	294 (35.3%) 486 (58.3%) 53 (6.4%)	45 (15.4%) 214 (73.3%) 33 (11.3%)	< 0.001
*Total cardiovascular calcium*				
Volume (cc)	837 [80, 3380]	494 [38, 2278]	2901 [843, 6691]	< 0.001
0 0–100 100–400 400–1000 > 1000	338 (30.0%) 359 (26.6%) 183 (16.2%) 127 (11.3%) 177 (15.7%)	293 (35.2%) 239 (28.7%) 129 (15.5%) 68 (8.2%) 104 (12.5%)	45 (15.4%) 61 (20.9%) 54 (18.5%) 59 (20.2%) 73 (25.0%)	< 0.001
Well-aerated lung volume, cc	2262 [1358, 3345]	2500 [1601, 3581]	1580 [918, 2481]	< 0.001
*Pneumonia, %*				
Absent Mild < 25% Moderate 25–50% Severe 50–75% Critical > 75%	13 (1.2%) 347 (30.8%) 397 (35.3%) 224 (19.9%) 144 (12.8%)	13 (1.6%) 304 (36.5%) 301 (36.1%) 138 (16.6%) 77 (9.2%)	0 (0.0%) 43 (14.7%) 96 (32.9%) 86 (29.5%) 67 (22.9%)	< 0.001
*Qualitative pneumonia features*				
Absent pneumonia GGO involving > 50% GGO and consolidation 50%/50% Consolidation > 50%	13 (1.2%) 623 (55.4%) 231 (20.5%) 258 (22.9%)	13 (1.6%) 438 (52.6%) 189 (22.7%) 193 (23.2%)	0 (0.0%) 185 (63.4%) 42 (14.4%) 65 (22.3%)	
MPAD, mm	27 [25, 30]	26 [24, 29]	29 [26, 31]	< 0.001
Paravertebral muscle density/Sarcopenia, HU	41 [32,48]	43 [-65, 128]	36 [-68, 61]	< 0.001
D11-D12 Bone density/Ostheoporosis, HU	128 [95, 165]	138 [11, 313]	111 [23, 250]	< 0.001
Liver density/fatty liver, HU	47 [36, 53]	47 [37, 53]	46 [32, 51]	0.04

The in-hospital binary outcome survivors (S) vs non-survivors (NS) was taken into consideration as the most reliable readout to assess patient’s outcome.

### Model development: from 24 to 5 variables model

Nine multivariate models were used for binary classification (Table 2). All models were trained including the 24 demographic, clinical and CT manually extracted variables and the Logistic regression model (glm) showed the highest AUC = 0.839 ± 0.009 (Table 2).

**Table 2:** AUC (area under the ROC curve) mean and standard deviation for nine model types, trained over the AI-SCoRE Var24 feature set. For each type, the optimal model was selected by caret over 10 × 5 runs M = 10 splits (n_train = 789).glm: generalized linear model; svmRadialSigma: Support Vector Machines with Radial Basis Function Kernel; rf: random forest; rf-bal: class-balanced random forest (sampling size at node equal to minority class for both classes); lda: Linear Discriminant Analysis; gbm: Stochastic Gradient Boosting; nb: Naive Bayes; C5.0: Ross Quinlan’s information gain tree; knn: k-Nearest Neighbors

Model	*mean*	*sd*
glm	0.8391	0.0090
svmRadialSigma	0.8365	0.0092
rf	0.8339	0.0077
rf-bal	0.8319	0.0098
lda	0.8298	0.0090
gbm	0.8285	0.0081
nb	0.8279	0.0066
C5.0	0.8007	0.0115
knn	0.7605	0.0145

Based on collinearity among variables and clinical redundancy we further focused on a subset of 13 variables (age, gender, COPD, diabetes, hypertension, oxygen saturation, creatinine, CRP, Liver steatosis, well-aerated lung volume, main pulmonary artery diameter, vertebral attenuation and total cardiovascular thoracic calcium). The testing of the 13-variable dataset in the same DAP confirmed the accuracy of the glm model (AUC = 0.840 ± 0.0093). In order to obtain a fast and unbiased prognostic score, we excluded the variables that could introduce biases, e.g., comorbidities, laboratory tests requiring long turnaround time and potentially affected by non-standardized reference values among different hospitals, all subjective and operator-dependent CT variables. Thus, the final model was tested on a reduced set of 5 variables (Var5): age, gender, oxygen saturation and two CT-derived variables (well-aerated parenchyma volume and total cardiovascular thoracic calcium volume) suitable for automatic extraction. The Var5 model had a mean AUC = 0.834 ± 0.007, very close to the AUC obtained on the larger sets with 24 and 13 variables. The glm models developed on the three feature sets were then compared on the test portions of the splits, with results consistent with the estimates on the internal tests (Tables S1-S2). In particular, an extensive round robin analysis of ROC curves by the Delong test [24, 25] supported the hypothesis that the three models were indistinguishable between feature sets and were statistically equivalent.

### Fully automatic condensed model

**Fig. 3:**
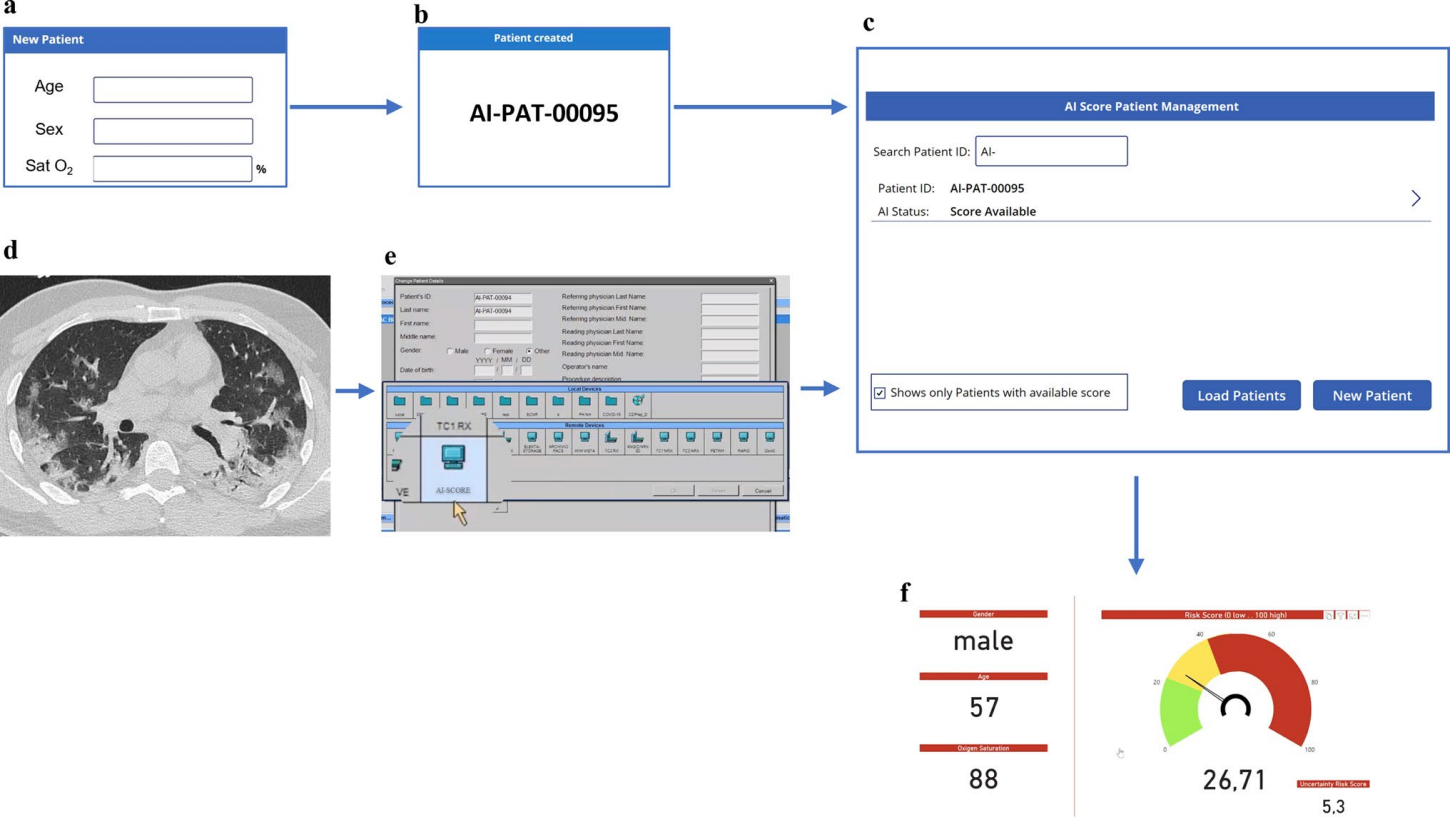
Example of AI-SCoRE computation. After patient’s age, sex and oxygen saturation are stored via an interface developed in PowerApp in Microsoft Teams environment (**a**), the system generates an anonymized patient ID (**b**). In parallel, patient’s anonymized chest CT images (**d**) can be uploaded on the platform via a connection node (**e**) in order to be automatically analyzed. In 15 min a pop up message is shown on the PowerApp alerting that a risk score 0–100 has been generated for the specific patient’s ID (**c**), together with the patient’s risk class (**f**) classified using color-coded graphs (green, yellow and red for low, medium and high risk)

A graphical and statistical summary of the set of quantitative features extracted from deep learning or threshold-based filters [8, 21–23] is reported in Fig. 3S. Alternative models were compared on internal and external tests with the same DAP (Table S3). The R5p model (i.e., Var5 glm model, with automatically extracted total cardiovascular calcium and WALV%_E) was the most accurate in internal tests (AUC = 0.837). A Delong test was again applied to estimate the impact of automated variables. No significant differences were found by pairwise evaluation over external tests among the R5p model and previously built models on Var5, Var13 and Var24 (Table S4). Coefficients for model R5p are reported in Table S5. The AUC estimated for the full first wave datasets was AUC = 0.842 (DeLong 95% CI: 0.816–0.867). The fully automated 5 variables model (R5p) has been selected for computing the “AI-SCoRE,” to predict mortality with a score ranging 0–100. A flowchart summarizing the development of the 5-feature automated model is provided in Fig. 1.

### Identification of risk classes

Classification tree (rpart implementation of CART [26]) was used to implement an automatic binning strategy in three classes with a maximum of 10% of false negatives in the low-risk bin (Fig. 1 on bottom right). A low specificity was instead accepted for the medium- and high-score groups. The binning method identified the AI-SCoRE interval (≥ 0, < 23) for the low-risk, (≥ 23, < 45) for the medium-risk and (≥ 45, ≤ 100) for the high-risk bin leading to a rate of mortality of 8%, 32% and 66% for each risk classes, respectively (Fig. 1 and Table 3).

**Table 3:** Contingency tables for wave 1 and wave 2 based on patients’ low, medium and high risk. *S* Survivors, *NS* non-survivors

	Wave 1			Wave 2		
	Proportion of total			Proportion of total		
Risk	Low	Medium	High	Low	Medium	High
Bin	≥ 0, < 23	≥ 23, < 45	≥ 45, ≤ 100	≥ 0, < 23	≥ 23, < 45	≥ 45, ≤ 100
S	0.15	0.51	0.07	0.56	0.11	0.18
NS	0.04	0.07	0.14	0.02	0.03	0.09
	Frequency			Frequency		
	Low	Medium	High	Low	Medium	High
S	579	172	82	119	24	39
NS	49	81	162	4	7	20

### Online platform development

**Fig. 4:**
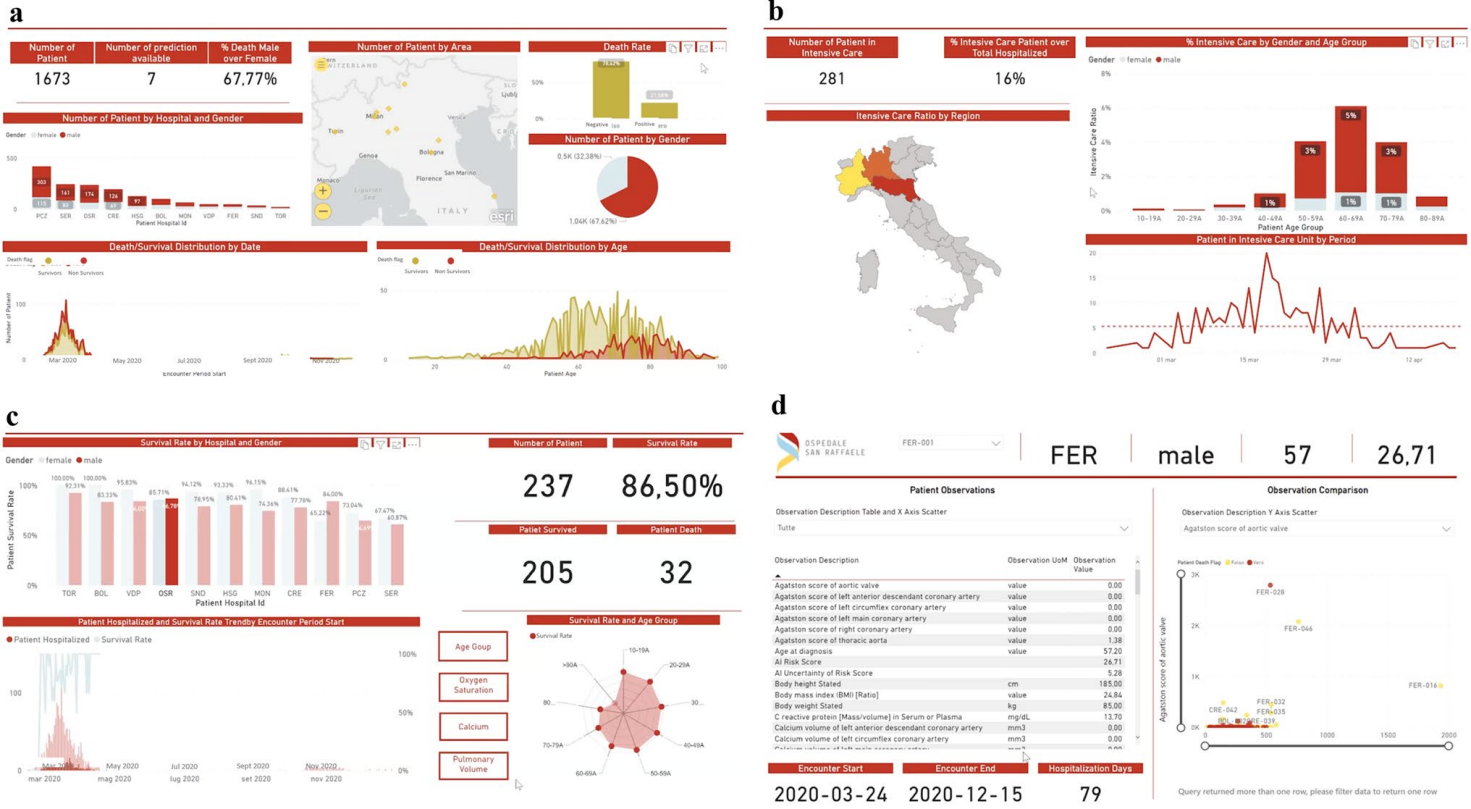
Patients data stored in PowerBI environment for extended research and statistical purposes. Different PowerBI dashboards have been generated in order to provide additional information about patients’ demographics (**a**), patients distribution in intensive care units (**b**) and patients’ survival rate according to hospitals, gender, clinical and radiological features (**c**) selected for the AI -SCoRE computation. In addition, a specific dashboard related to all parameters collected for each patient has been added (**d**), with the aim of possibly extending the research beyond COVID-19 and finding possible correlations and trends among parameters

A user-friendly interface has been developed in a PowerApp environment, enabling the AI-SCoRE platform to be accessed via both PC desktop and mobile app. Given the patient’s age, gender and oxygen saturation, the system generates an anonymized patient ID, which is used to anonymize the CT images. The anonymized CT images can be uploaded via a connection node and automatically analyzed. In 6 ± 2 min, the system autonomously generates a risk score (AI-SCoRE 0–100) and corresponding color-coded risk class (green, orange and red for low, intermediate and high risk, respectively), which is notified by a popup message (Fig. 3). The AI-SCoRE platform also provides additional functions (Fig. 4), including fast access to demographic, clinical, laboratory test, imaging and outcome data. The platform also includes the Power BI “Question & Answer” functionality, enabling interactive requests and fast retrieval of data.

### Prospective validation of AI-SCORE on wave 2 cohort

The second wave cohort showed lower age (65 y.o., IQR [55, 76], p = 0.004) and a lower oxygen saturation level (91 y.o., IQR [86, 94]; *p* = 0.003), and a lower rate of mortality with 31 (14.6%) deceased subjects (Fisher's exact test *p* < 0.001), compared to the first wave cohort.

On second wave, AI-SCoRE had an AUC of 0.808 (DeLong 95% CI: 0.740–0.877) with 123 patients (58%) assigned to the low-risk class. The mortality rate was 1.8% for low-risk class, 23% for medium-risk class and 34% for high-risk class (Fig. 1 on bottom left and Table 3).

**Fig. 5:**
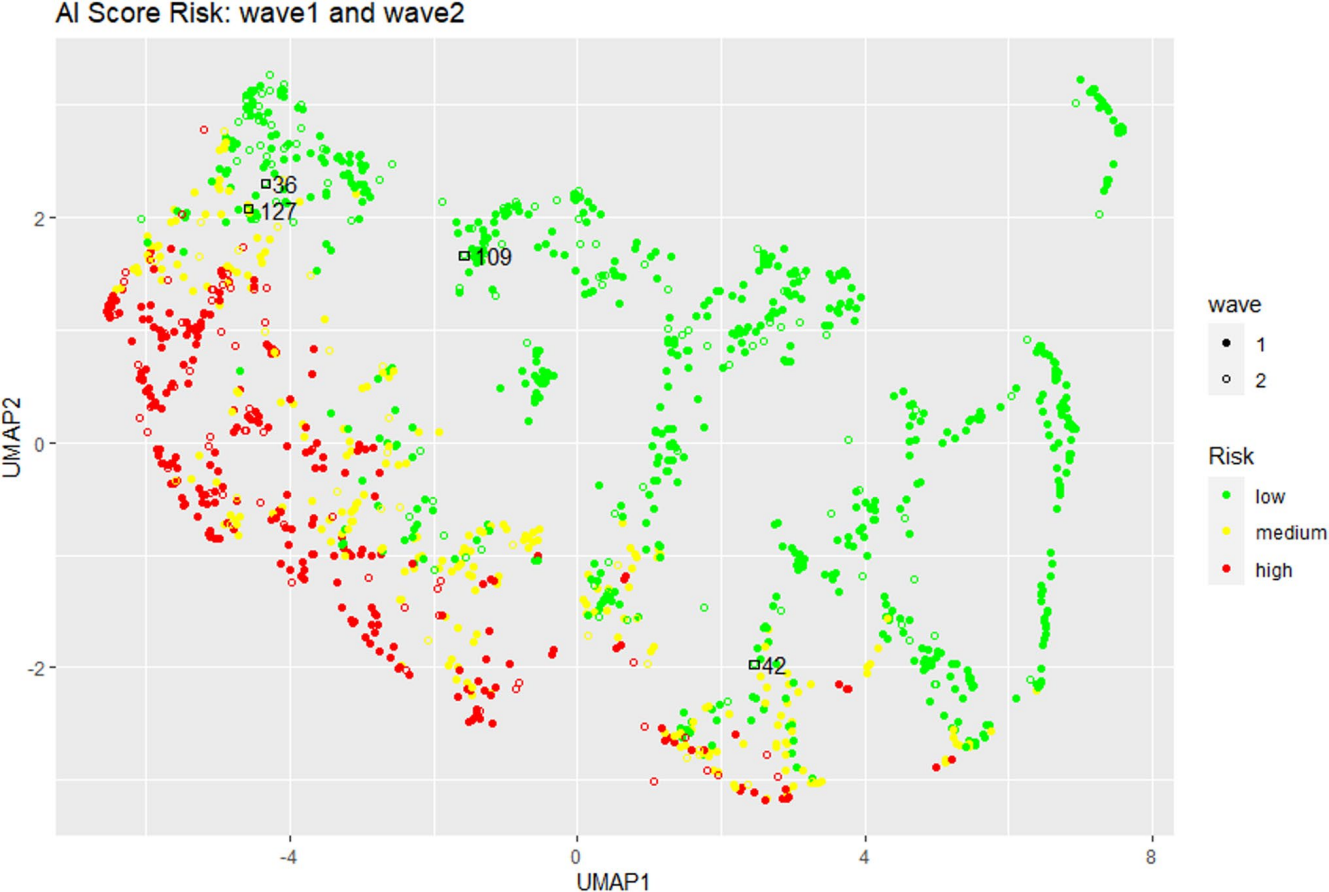
Uniform Manifold Approximation and Projection (UMAP) for the AI-SCoRE risk classification on wave 1 (filled dots) and wave 2 (empty dots). The UMAP projection was computed on wave 1 and applied in inference on wave 2. Empty black squares indicate the four misclassified samples in wave 2 (36; 42; 109; 127)

**Fig. 6:**
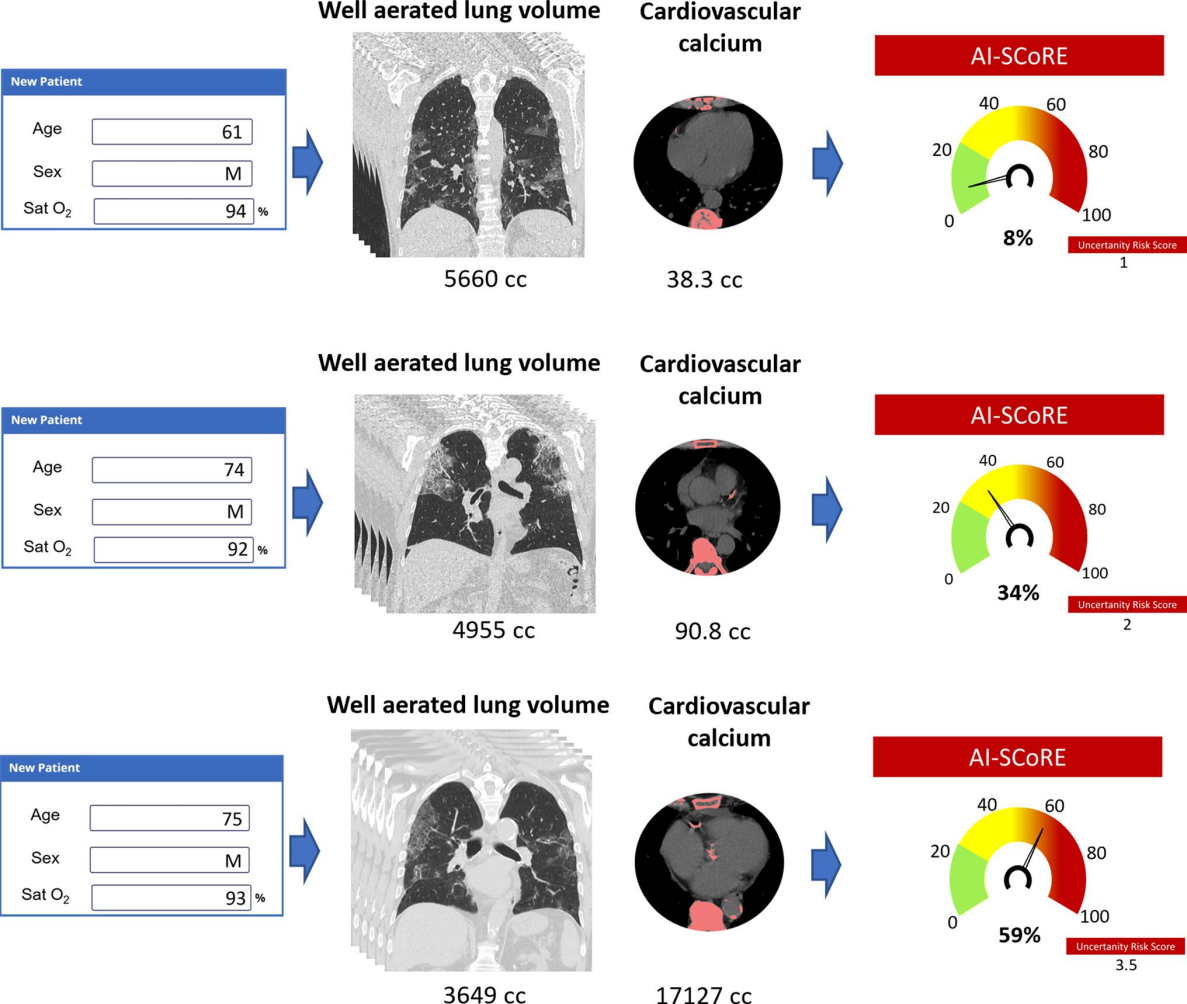
Exemplifying cases of AI-SCoRE prospective validation. After the introduction of clinical data including age, sex and oxygen saturation, DICOM chest CT images are anonymously uploaded in the platform through a connection node and the volume of well-aerated lung volume and the total cardiovascular thoracic calcium computed. Then, in few minutes, the platform generates patient’s “AI-SCoRE” risk score with green color for low risk value (≥ 0, < 23), yellow for moderate risk (≥ 23, < 45) and red for high risk value (≥ 45, ≤ 100). On top is reported the case of a patient classified at low risk, in the middle a patients classified at moderate risk and in the bottom a case of a patients classified at high risk. The case on top of the image is a 61-year-old man presented to the emergency department for fever and cough from 10 days. In ambient air oxygen saturation was 94%. Chest CT scanning was obtained and the resulting AI-SCoRE was 8%. The patient was discharged after 12 days of hospitalization. In the middle, a 74-year-old man presented to the emergency department for fever and anosmia from 6 days. Oxygen saturation was 92% and after integration of age, sex, oxygen saturation and chest CT images on the platform, the AI-SCoRE was 34% (moderate risk). In few days, patients had severe desaturation and noninvasive ventilation was required for 15 days. After 20 days, the patient was discharged. Finally, in the bottom a 75-year-old man presented to the emergency department for fever and cough from 5 days. Oxygen saturation was 93%, and AI-SCoRE showed high risk (59%). The patient had a progressive worsening of oxygen saturation requiring high-flow oxygen therapy and noninvasive ventilation, but unfortunately he died for sudden cardiac death 14 days later

All the 4 patients who died although classified in the low risk were affected by mild COVID-19, but presented severe comorbidities with an already reduced expectancy of life and/or on immunosuppressive chronic therapy (patient 36: mixed connective tissue disease treated with rituximab and cyclophosphamide; patient 42: rheumatoid arthritis treated with methotrexate and low-dose steroids; patient 109: chronic lymphocytic leukemia, RAI II, on ibrutinib treatment; and patient 120: systemic amyloidosis, previous liver transplantation and chronic heart failure). Excluding these 4 patients due to the preexisting critical clinical conditions and immunocompromised status that place these patients at high risk regardless of any specific score for COVID-19, the AI-SCoRE correctly classified all the patients at low risk of the second wave cohort (Fig. 5). Exemplifying cases of AI-SCoRE prospective validation are reported in Fig. 6.

## Discussion

Implementing rapid and effective automated tools to profile COVID-19 patients with respect to their risk of death or hospitalization represents a fundamental challenge for a better allocation of patients and health resources. General clinical risk scores used in the emergency department, such as SOFA and MEWS, when applied to COVID‐19 infection, unfortunately lack adequate sensitivity and specificity to predict mortality associated with COVID‐19 infection [27, 28].

Therefore, in the last months several attempts to improve patients risk stratification was performed developing clinical risk scores also based on ML algorithms. However, the real clinical applicability of the proposed methods is unclear, mainly for methodological issues concerning scarce quality of raw data, heterogeneity and lack of standardization of collected variables, biases in outcome definition and unclear resolution of bias [6, 29].

In order to fulfill the clinical need, overcoming the aforementioned methodological limitation, we have developed the fully automatic AI-SCoRE platform, able to provide a patient risk score in a 0–100 scale, based on the evaluation of only five variables: two demographic data (age and gender), one standardized clinical data of very fast and easy measurement (oxygen saturation) and two quantitative imaging features automatically extracted by a conventional non-contrast chest CT scan (the well-aerated lung volume and the total cardiovascular thoracic calcium).

Outcome was defined as survival, considered as the most reliable data during COVID-19 pandemic, for low reliability of information about oxygen therapy due to fragmented collection of data in emergency and for bias in deployment of treatment (e.g., ICU access) according to hospital resources and pandemic phases.

The AI-SCORE was developed on a retrospective series of 1125 patients referred to 16 Italian hospitals in a limited time period and prospectively validated on 214 consecutive patients during the second wave.

This model showed good performances in the prediction of patient’s outcome in both the first and second waves (AUC = 0.842 and AUC = 0.808), despite the significant improvement of treatment during second wave with subsequent reduction in the overall mortality rate. Notably, the AI-SCORE showed a non-inferior performance compared to models (Vars24 and Vars13) including a larger set of patients’ clinical and laboratory test features, highlighting its clinical value and applicability.

The AI-SCoRE algorithm and platform was able to identify the three risk classes, with only 1.8% of patients misclassified as low risk in the external prospective validation on second wave, all of them with preexisting severe condition determining a strongly reduced expectancy of life.

Our final algorithm included common demographics as age and sex [3, 4, 8, 15], and oxygen saturation, which are all well-recognized predictors of patients’ outcome and crucial parameter to guide patient’s treatment and management [1]. The AI score platform integrates these parameters with chest CT metrics automatically extracted from the entire volume of lung parenchyma and thoracic vessels. The automatic volumetric analysis of lung involvement guarantees a more realistic and accurate measurement of pneumonia severity score [30], in comparison with analysis of isolated 2D slices or even 2D patches used in some previous studies [6], as well as in comparison with semiquantitative score derived from radiologist reading [4, 18], which are affected by limited panoramicity or reader subjectivity.

Moreover, the use of chest CT images instead of XR images guarantees higher sensitivity in the identification of lung parenchyma involvement, with full consideration of slight inflammatory changes [31], and the possibility of a deeper patients’ phenotyping through the quantification of calcium deposits in cardiac valves and thoracic vessels [32].

AI-SCoRE is the first ML COVID-19 risk model integrating cardiovascular calcium. This provides a more comprehensive assessment of patients’ risk. Coronary calcium score is a marker of coronary artery disease and is an established independent predictor of mortality and cardiovascular events in the general population [33]. It was associated with critical illness, adverse major cardiovascular events and death in COVID-19 patients [10, 11, 34, 35]. Total thoracic cardiovascular calcium, which includes also aortic valve and thoracic aorta calcium, resulted a stronger predictor of prognosis in COVID-19 patients if compared to coronary calcium score alone, suggesting that total calcium provides a more comprehensive assessment of systemic atherosclerosis and cardiovascular senescence and left ventricle overload [36]. Its prognostic value may originate from several factors. First of all, endothelium is a target of SARS-CoV-2 infection and diffuse endothelitis has a pivotal role in determining multiple organ damage, hence the chronic endothelial dysfunction and endothelial inflammatory state occurring in atherosclerosis may increase susceptibility to COVID-19 systemic injury [10, 37, 38].

The development of AI-SCoRE was based on a clinical machine learning perspective. This consisted in a first step based on pure ML approach in which interactions between clinical and imaging covariates, and patient outcomes were obtained in a fully data-driven manner. Then, according to recent criticisms about limited predictive power of complete data-driven approaches to COVID-19 [6], a further clinical-driven reduction of the variables was performed with the exclusion of comorbidities, laboratory tests and subjective measurements, potentially affected by limited generalizability due to challenge collection in emergency, inter-laboratories differences in reference values or inter-reader variability for manual measurement.

Differently from most of previous predictive models [3, 4, 6, 30], in our study the outcome was defined as patients survival, considered as the most reliable endopoint during COVID-19 pandemic, due to scarce reliability of other endpoints affected by local protocols and hospital resources.

AI-SCoRE requires only a few and easy to be collected variables, also for poorly equipped hospitals facing a pandemic in a overwhelmed condition. This architecture of the algorithm allowed to avoid missed data, differently from most of previously developed algorithms in which from 30% to more than 50% of patients enrolled did not have all required values collected [18] with imputation of missing data often used [18, 39]. AI-SCoRE model was developed and validated only on a complete dataset, avoiding imputation of missing data, with subsequent more realistic performance metrics and higher applicability in clinical practice.

An important novelty in our procedure is the considerable reduction of time to diagnosis consistent with the urgent public health needs of optimizing health resources. AI-SCoRE may support clinical decision making (home care, mobile hospital quarantine, hospitalization or access to ICU) at hospital admission.

Similarly to previous studies on COVID-19, one limitation of our study consists in potential heterogeneity of data collected in a short time interval from multiple centers in an emergency setting. However, the multicenter approach is mandatory for reducing biases and increase generalizability of the prediction model. Notably, CT parameters have been centrally analyzed in the first step of the study and fully automatically extracted in the final step, significantly reducing the risk of bias. Moreover, validation on second wave cohort, under different public health conditions, confirmed the effectiveness of AI-SCoRE in prediction of patients’ outcome also with optimized treatment.

Although the vaccine significantly reduces the infection rate and COVID-19 severity, the variability of adherence to vaccination policy with persistent spread of infection in non-vaccinated people suggests the potential usefulness of AI-SCoRE platform to improve the allocation of resources based on patients’ risk stratification.

## Supplementary Information

Below is the link to the electronic supplementary material.

Supplementary file1 (DOCX 1085 kb)

## Abbreviations

AI: Artificial intelligence
AI-SCoRE: Artificial intelligence – Sars Covid prognostic risk evaluation
AUC: Area under the curve
CT: Computed tomography
DAP: Data analysis plan
ML: Machine learning
ROC: Receiver operating curve

## References

[1] Organization World Health (2020) WHO Coronavirus (Covid19). In: World Health Organization. https://covid19.who.int. Accessed 23 Dec 2021

[2] Cummings MJ, Baldwin MR, Abrams D (2020). Epidemiology, clinical course, and outcomes of critically ill adults with COVID-19 in New York City: a prospective cohort study. The Lancet.

[3] Ciceri F, Castagna A, Rovere-Querini P (2020). Early predictors of clinical outcomes of COVID-19 outbreak in Milan, Italy. Clin Immunol.

[4] Liang W, Liang H, Ou L (2020). Development and validation of a clinical risk score to predict the occurrence of critical illness in hospitalized patients with COVID-19. JAMA Intern Med.

[5] Patel D, Kher V, Desai B (2021). Machine learning based predictors for COVID-19 disease severity. Sci Rep.

[6] Roberts M, Driggs D, Thorpe M (2021). Common pitfalls and recommendations for using machine learning to detect and prognosticate for COVID-19 using chest radiographs and CT scans. Nat Mach Intell.

[7] Palmisano A, Scotti GM, Ippolito D (2021). Chest CT in the emergency department for suspected COVID-19 pneumonia. Radiologia Medica.

[8] Esposito A, Palmisano A, Cao R (2021). Quantitative assessment of lung involvement on chest CT at admission: impact on hypoxia and outcome in COVID-19 patients. Clin Imaging.

[9] Loffi M, Regazzoni V, Toselli M (2021). Incidence and characterization of acute pulmonary embolism in patients with SARSCoV-2 pneumonia: a multicenter Italian experience. PLoS ONE.

[10] Giannini F, Toselli M, Palmisano A (2021). Coronary and total thoracic calcium scores predict mortality and provides pathophysiologic insights in COVID-19 patients. J Cardiovasc Comput Tomogr.

[11] Scoccia A, Gallone G, Cereda A (2021). Impact of clinical and subclinical coronary artery disease as assessed by coronary artery calcium in COVID-19. Atherosclerosis.

[12] Bertini M, D’aniello E, Cereda A (2021). The combination of chest computed tomography and standard electrocardiogram provides prognostic information and pathophysiological insights in COVID-19 pneumonia. J Clin Med.

[13] Sticchi A, Cereda A, Toselli M (2021). Diabetes and mortality in patients with COVID-19: Are we missing the link?. Anatol J Cardiol.

[14] Conte C, Esposito A, de Lorenzo R (2021). Epicardial adipose tissue characteristics, obesity and clinical outcomes in COVID-19: A post-hoc analysis of a prospective cohort study. Nutr Metab Cardiovasc Dis.

[15] Esposito A, Palmisano A, Toselli M (2021). Chest CT–derived pulmonary artery enlargement at the admission predicts overall survival in COVID-19 patients: insight from 1461 consecutive patients in Italy. Eur Radiol.

[16] Ufuk F, Demirci M, Sagtas E (2020). The prognostic value of pneumonia severity score and pectoralis muscle Area on chest CT in adult COVID-19 patients. Eur J Radiol.

[17] Li L, Qin L, Xu Z (2020). Using artificial intelligence to detect COVID-19 and community-acquired pneumonia based on pulmonary CT: evaluation of the diagnostic accuracy. Radiology.

[18] Liang W, Yao J, Chen A (2020). Early triage of critically ill COVID-19 patients using deep learning. Nat Commun.

[19] Shi L, Campbell G, Jones WD (2010). The Microarray Quality Control (MAQC)-II study of common practices for the development and validation of microarray-based predictive models. Nat Biotechnol.

[20] Kuhn M, Johnson K (2013). Applied predictive modeling. Appl Pred Model.

[21] Hofmanninger J, Prayer F, Pan J (2020). Automatic lung segmentation in routine imaging is primarily a data diversity problem, not a methodology problem. Eur Radiol Exp.

[22] Milletari F, Navab N, Ahmadi SA (2016) V-Net: fully convolutional neural networks for volumetric medical image segmentation. In: proceedings 2016 4th international conference on 3D vision, 3DV 2016 pp. 565–571

[23] NVIDIA Clara COVID-19 Collection. https://ngc.nvidia.com/catalog/models/nvidia:clara_train_covid19_ct_lesion_seg. Accessed 23 Dec 2021

[24] DeLong ER, DeLong DM, Clarke-Pearson DL (1988). Comparing the areas under two or more correlated receiver operating characteristic curves: a nonparametric approach. Biometrics.

[25] Robin X, Turck N, Hainard A (2011). pROC: An open-source package for R and S+ to analyze and compare ROC curves. BMC Bioinf.

[26] Therneau T, Atkinson B, Ripley B (2015) Rpart: Recursive partitioning and regression trees. R package version 4.1–00. http://CRAN.R-project.org/package=rpart. https://cran.r-project.org/package=rpart

[27] Zhou F, Yu T, Du R (2020). Clinical course and risk factors for mortality of adult inpatients with COVID-19 in Wuhan, China: a retrospective cohort study. The Lancet.

[28] Tang X, Du RH, Wang R (2020). Comparison of hospitalized patients with ARDS caused by COVID-19 and H1N1. Chest.

[29] The Lancet Digital Health (2021). Artificial intelligence for COVID-19: saviour or saboteur?. Lancet Dig Health.

[30] Wang R, Jiao Z, Yang L (2022). Artificial intelligence for prediction of COVID-19 progression using CT imaging and clinical data. Eur Radiol.

[31] Choi H, Qi X, Yoon SH (2020). Extension of coronavirus disease 2019 on chest ct and implications for chest radiographic interpretation. Radiol Cardiothorac Imaging.

[32] Cereda A, Allievi L, Palmisano A (2022). Systematic review and meta-analysis on coronary calcifications in COVID-19. Emerg Radiol.

[33] Budoff MJ, Young R, Burke G (2018). Ten-year association of coronary artery calcium with atherosclerotic cardiovascular disease (ASCVD) events: the multi-ethnic study of atherosclerosis (MESA). Eur Heart J.

[34] Zimmermann GS, Fingerle AA, Müller-Leisse C (2020). Coronary calcium scoring assessed on native screening chest CT imaging as predictor for outcome in COVID-19: an analysis of a hospitalized German cohort. PLoS ONE.

[35] Luchian M-L, Lochy S, Motoc A (2021). Prognostic value of coronary artery calcium score in hospitalized COVID-19 patients. Front Cardiovas Med.

[36] Pawade T, Clavel MA, Tribouilloy C (2018). Computed tomography aortic valve calcium scoring in patients with aortic stenosis. Circul Cardiovas Imaging.

[37] Nishiga M, Wang DW, Han Y (2020). COVID-19 and cardiovascular disease: from basic mechanisms to clinical perspectives. Nat Rev Cardiol.

[38] Evans PC, Rainger G, Mason JC (2020). Endothelial dysfunction in COVID-19: a position paper of the ESC working group for atherosclerosis and vascular biology, and the ESC council of basic cardiovascular science. Cardiovasc Res.

[39] Zhu JS, Ge P, Jiang C (2020). Deep-learning artificial intelligence analysis of clinical variables predicts mortality in COVID-19 patients. J Am Coll Emerg Phys Open.

