# Supplementary material for: AI-SCoRE (artificial intelligence-SARS CoV2 risk evaluation): a fast, objective and fully automated platform to predict the outcome in COVID-19 patients
Source: Radiol Med. 2022 Aug 29;127(9):960–72. doi: 10.1007/s11547-022-01518-0 (PMC9423702; doi:10.1007/s11547-022-01518-0)
Supplement: Supplementary file 1 — Supplementary file1 (DOCX 1085 kb) [file 11547_2022_1518_MOESM1_ESM.docx]

**SUPPLEMENTARY METHODS**

Demographic, clinical, and and lab test data were collected from 1575 consecutive patients, admitted to the ED of 16 hospitals in Italy ) for COVID-19 confirmed diagnosis at RT-PCR, during the first wave of pandemic (from February 16th to April 29th, 2020).

List of partecipant hospitals:

1. IRCCS Ospedale San Raffaele, Milano
2. Maria Cecilia Hospital - GVM Care & Research, Cotignola;
3. ASST Bergamo Est, Seriate;
4. ASST Monza Ospedale San Gerardo;
5. Centro cardiologico Monzino, Milano;
6. Azienda Ospedaliero-Universitaria di Parma;
7. Ospedale Guglielmo da Saliceto, Piacenza;
8. Ospedale Maggiore - Bologna;
9. Azienda Ospedaliero-Universitaria di Ferrara, Ferrara
10. ASST Papa Giovanni XXIII, Bergamo;
11. ASST Valtellina and Alto Lario, “Eugenio Morelli Hospital";
12. ASST Cremona;
13. ASST Lecco Merate;
14. ASL Città di Torino, Ospedale San Giovanni Bosco;
15. Casa di Cura Villa dei Pini, Civitanova Marche;
16. ICC - Istituto Clinico Casalpalocco, Roma;

Collected clinical parameters were: (i) demographic characteristics (age, gender), (ii) cardiovascular risk factors (body mass index, hypertension, diabetes, history of coronary artery disease and revascularization), (iii) oxygen saturation, (iv) comorbidities (chronic lung disease, chronic kidney disease, active malignancy, peripheral artery disease), (v) laboratory tests (hemoglobin, white blood cells count, creatinine, high sensitivity troponin I (HS-TnI),lactate dehydrogenase (LDH), C-reactive protein (CRP), Interleukin-6 and D-dimer), (vi)  Major adverse cardiac and cerebrovascular events (MACCE) including myocardial infarction, stroke, pulmonary thromboembolism, peripheral ischemia, (vii) oxygen therapy (no therapy, oxygen, non-invasive ventilation, invasive ventilation).

The in-hospital outcome (death vs discharge) for each patient was recorded. Data collection was concluded on June 30^th^, 2020.

All patients underwent non-contrast chest CT scan with at least 16 detector rows within 72 hours from admission. Non-contrast chest CT were collected and analyzed in the coordinating center (IRCCS San Raffaele Hospital) by a radiologist with 10 years of experience in cardiothoracic imaging, using a dedicated semiautomatic software (IntelliSpace Portal v. 8.0, Philips Medical Systems).

The CT parameters analyzed were:

1. Parameters of lung respiratory reserve: (i) well-aerated lung volume in mL, (ii) percentage of pneumonia extension according to Bernheim et al.[1], (iii) number of lung lobes involved by pneumonia, (iv) qualitative features of pneumonia lesion “score 0 for absent pneumonia, score 1 for prevalent ground-glass opacities (GGOs), score 2 for prevalent consolidation, and score 3 for GGOs and consolidation equally represented, v) pneumonia pattern (1:central, 2: peripheral, 3: diffuse), (vi) presence or not of pleural effusion;

2. Parameters of cardiovascular atherosclerosis: (i) presence of coronary calcium or stent, (ii) number of coronary vessels with calcium, (iii) per-vessel and per-patients Agatston score and calcium volume (cc), (iv) aortic valve Agatston score and calcium volume (cc), (v) thoracic aorta Agatston score and calcium volume (cc), (vi) cardiac chamber dimensions (left and right ventricle, left and right atrium);

3. Parameters of pulmonary hypertension: (i) main pulmonary artery diameter, (ii) left pulmonary artery diameter, (iii) right pulmonary artery diameter, (iv) pulmonary artery diameter-aortic diameter ratio;

4. Parameters of fragility: (i) sarcopenia (mean HU ± stDev of both paravertebral muscles at D12 level), (ii) osteoporosis (mean HU ± stDev of D12), (iii) liver steatosis (mean HU ± stDev of both lobes).

**Data extraction, quality assurance and processing**

Demographics, clinical and laboratory data were extracted by expert clinicians of each hospital from electronic medical records.

Retrospective data were recorded into a computerized database and cross-checked in the coordinating center (IRCCS San Raffaele) by a multidisciplinary team including clinicians, radiologists, biostatisticians and computer scientists. To ensure adequate data-quality, data cleansing has been implemented to avoid major errors and inconsistencies, to standardize measurement, to verify type of missingness in variables and missing at random (MAR). The in-hospital binary outcome survivors (S) vs non-survivors (NS) was taken into consideration as the most reliable measurable read-out to assess each patient's disease evolution. Final cohort included 1125 patients and 24 variables.

**Automatic extraction of lung and calcium quantitative CT features**

*Well-aerated lung volume and pneumonia features.*

In order to automatically extract quantitative pneumonia features from CT images, we used a combination of two pre-trained deep-learning models. Complete lung masks were obtained using the publicly available R231 model [2]. The model is a 2D U-net operating on individual slices, which was trained on a dataset representative of diverse imaging protocols and disease states.

A second pre-trained segmentation model was employed to extract regions showing radiological signs of COVID-19. The model is available as part of the NVIDIA Clara COVID-19 Collection[3]. The architecture is a volumetric U-net, or V-net [4] trained on a CT series of 913 independent subjects with expert-provided region annotations. The CT series are resampled to 0.8x0.8x5mm and intensity is clipped in the [-1000, 500] HU range. Out of the whole series, the model is fed a sliding ROI of size 384x384x32 to produce a volumetric segmentation of the COVID-19 related regions, which include GGO and consolidations. The ratio between the volume of the COVID-19 pneumonia and the volume of the lung mask is computed. In order to guarantee that the pneumonia mask is contained in the lung mask for the calculation to be correct, the pneumonia mask is itself masked with the lung mask prior to taking the ratio.

In order to enrich the description of COVID-19 lesions beyond what extracted from the pneumonia model, the relative fractions of voxels within the lung mask corresponding to the HU intervals for GGO (-780, -570), semi-consolidation (-570, -290) and consolidation (≥ -290) proposed by Esposito et al. [5] were computed and used as additional features. Based on these threshold values the following parameters were calculated well-aerated lung volume (WALV%_E), ground glass opacities (GGO%_E), semi-consolidations (SC%_E), consolidations, and overall interstitial involvement (GGO-SC%_E).

*Total cardiovascular thoracic calcium.* An estimate of the total cardiovascular thoracic volume including coronary arteries, aortic valve and thoracic aorta calcium was obtained using a multistep approach. First, all voxels above 130 HU were extracted and a morphological closing operation was applied on the resulting binary mask. A connected component filter was then applied and thus extracted regions were sorted by their total volume in order to identify bones as the most voluminous ones. A morphological closing operation of a dilation operation of that mask was performed to isolate a bone mask. That mask was then subtracted from the mask obtained by thresholding the original voxels above 130 HU. The resulting mask underwent a morphological opening operation to obtain the final calcium mask. Last, the components of the calcium mask located in the region of space between the lung masks were extracted. Automatic estimator strongly correlates with the manual segmentation (R=0.844 p=2.2e-16), as resulting from an analytical validation experiment with a robust regression model (PaBablok method).

**REFERENCES**

1. Bernheim A, Mei X, Huang M, et al (2020) Chest CT findings in coronavirus disease 2019 (COVID-19): Relationship to duration of infection. Radiology 295:685–691

2. Hofmanninger J Automated lung segmentation in CT under presence of severe pathologies. https://github.com/JoHof/lungmask. Accessed 23 Dec 2021

3. NVIDIA Clara COVID-19 Collection. https://ngc.nvidia.com/catalog/models/nvidia:clara_train_covid19_ct_lesion_seg. Accessed 23 Dec 2021

4. Milletari F, Navab N, Ahmadi SA (2016) V-Net: Fully convolutional neural networks for volumetric medical image segmentation. Proceedings - 2016 4th International Conference on 3D Vision, 3DV 2016 565–571. https://doi.org/10.1109/3DV.2016.79

5. Esposito A, Palmisano A, Cao R, et al (2021) Quantitative assessment of lung involvement on chest CT at admission: Impact on hypoxia and outcome in COVID-19 patients. Clinical Imaging 77:194–201. https://doi.org/10.1016/j.clinimag.2021.04.033

**SUPPLEMENTARY FIGURES:**

**Figure S1:** Analytical validation of the calcium radiomics estimator. Method1: standard measure; Method2: radiomics estimate.


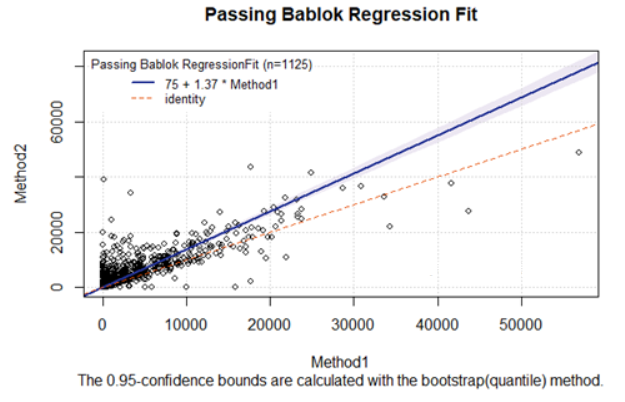


**Figure S2:** Automatic segmentation of lung involvement and cardiovascular calcification from non contrast chest CT scan. In A is reported the original chest CT scan. In B automatic segmentation of cardiovascular calcium (in red) based on threshold method. C: lungs segmented with the deep learning model; D: overall pneumonia involvement segmented by NVIDIA Clara model; From E to H is showed lung pneumonia features segmentation by using the thresholding approach: consolidations in E; semi-consolidations (SC_E) in F; ground-glass opacities (GGO_E) in G; well-aerated lung volume (WALV_E) in H.


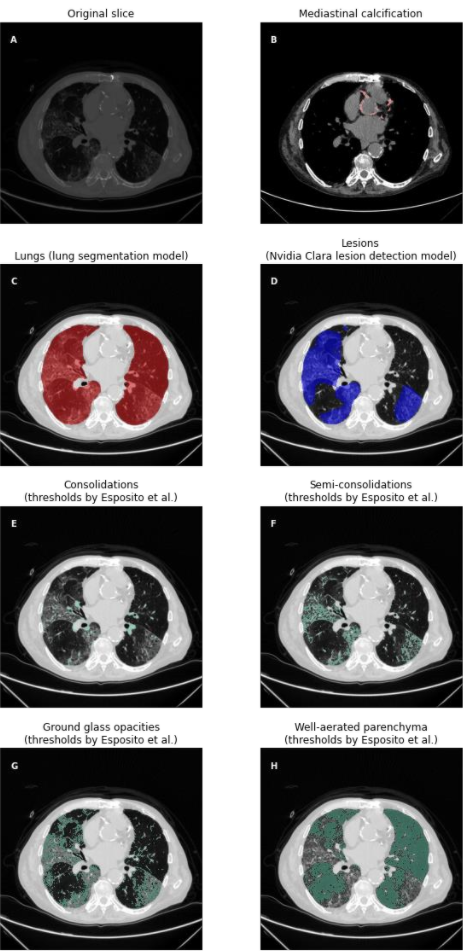


**Figure S3.** Statistical distribution of model candidate predictors. Definitions: SatO2: oxygen saturation in ambient Air; Tot calcium: total cardiovascular thoracic calcium; Vol R231: Non consolidated lung volume from model R231; pneumonia_C: Percentage of lung involvement according to Clara V-net segmentation model; WALV%_E: percentage of well-aerated lung parenchyma from threshold method; GGO-SC_E: percentage of overall interstitial involvement (GGO+ semiconsolidation); Outcome: S survived (cyan), NS not-survived (red).


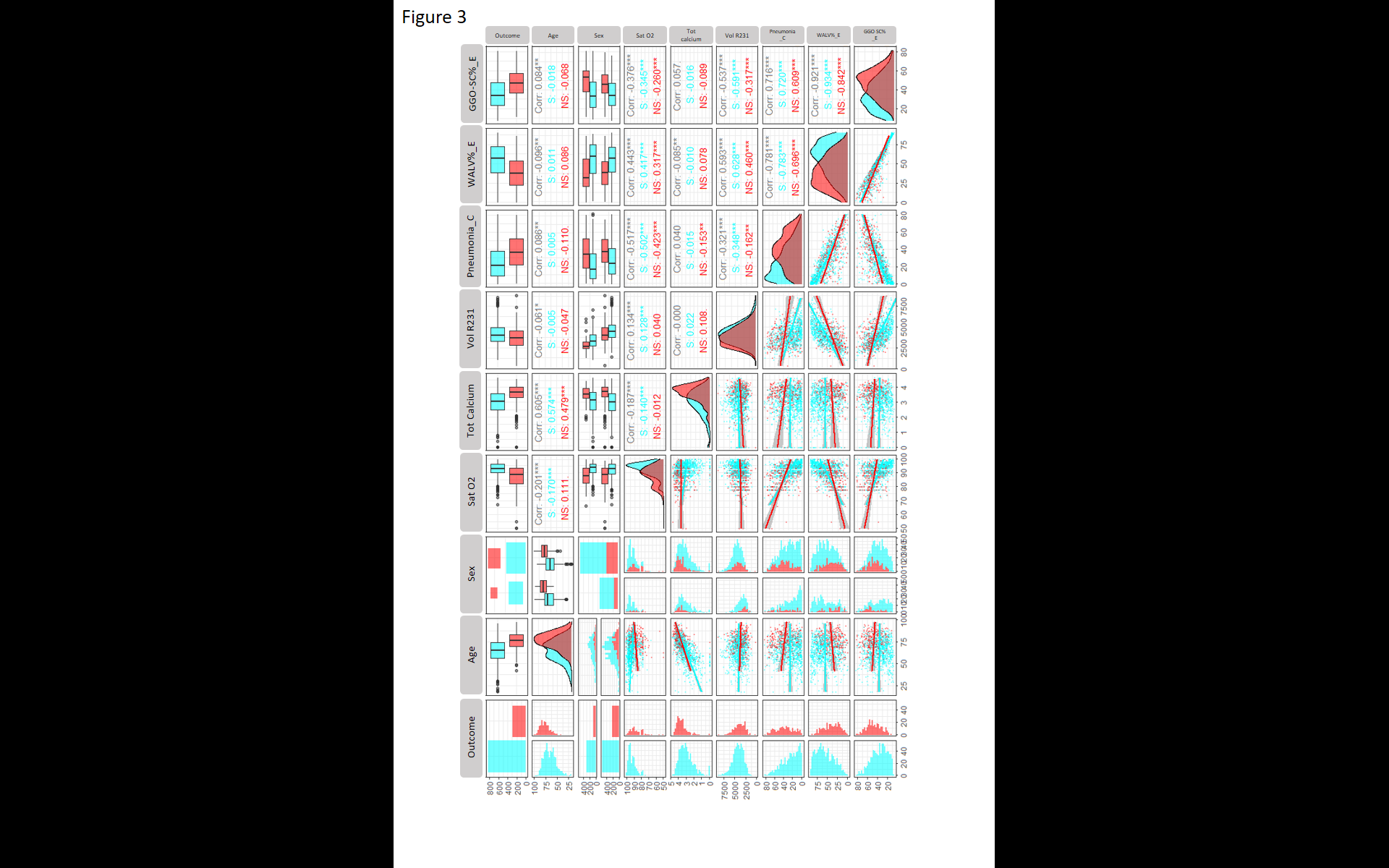


**SUPPLEMENTARY TABLES:**

**Table 1S.** Wave1 dataset. AUC on external test for glm on the v5, v13 and v24 feature sets for M=10 splits (n_test=336).

| **Model** | **X1** | **X2** | **X3** | **X4** | **X5** | **X6** | **X7** | **X8** | **X9** | **X10** | **mean** | **SD** |
| --- | --- | --- | --- | --- | --- | --- | --- | --- | --- | --- | --- | --- |
| **glm V5** | 0.826 | 0.828 | 0.833 | 0.828 | 0.839 | 0.814 | 0.813 | 0.824 | 0.799 | 0.806 | 0.821 | 0.013 |
| **glm V13** | 0.833 | 0.838 | 0.849 | 0.831 | 0.856 | 0.833 | 0.814 | 0.839 | 0.805 | 0.790 | 0.829 | 0.020 |
| **glm V24** | 0.837 | 0.835 | 0.853 | 0.835 | 0.855 | 0.840 | 0.819 | 0.841 | 0.804 | 0.797 | 0.831 | 0.019 |

**Table 2S.** Delong pairwise comparison of the 10 models built on Var5, Var13, Var24 (p-values).

|  | **v24_1** | **v24_2** | **v24_3** | **v24_4** | **v24_5** | **v24_6** | **v24_7** | **v24_8** | **v24_9** | **v24_10** |
| --- | --- | --- | --- | --- | --- | --- | --- | --- | --- | --- |
| **v5_1** | 0.289 | 0.708 | 0.477 | 0.870 | 0.371 | 0.826 | 0.743 | 0.691 | 0.559 | 0.356 |
| **v5_2** | 0.871 | 0.363 | 0.514 | 0.926 | 0.399 | 0.882 | 0.682 | 0.739 | 0.502 | 0.311 |
| **v5_3** | 0.997 | 0.884 | 0.055 | 0.951 | 0.500 | 0.990 | 0.578 | 0.861 | 0.417 | 0.255 |
| **v5_4** | 0.866 | 0.756 | 0.520 | 0.747 | 0.407 | 0.876 | 0.699 | 0.737 | 0.522 | 0.329 |
| **v5_5** | 0.867 | 0.974 | 0.770 | 0.819 | 0.024 | 0.851 | 0.476 | 0.999 | 0.338 | 0.203 |
| **v5_6** | 0.577 | 0.479 | 0.293 | 0.628 | 0.220 | 0.074 | 0.991 | 0.471 | 0.783 | 0.524 |
| **v5_7** | 0.570 | 0.476 | 0.296 | 0.619 | 0.225 | 0.573 | 0.964 | 0.468 | 0.810 | 0.551 |
| **v5_8** | 0.789 | 0.684 | 0.463 | 0.841 | 0.362 | 0.798 | 0.781 | 0.065 | 0.597 | 0.386 |
| **v5_9** | 0.340 | 0.270 | 0.151 | 0.379 | 0.111 | 0.338 | 0.688 | 0.269 | 0.542 | 0.817 |
| **v5_10** | 0.460 | 0.380 | 0.233 | 0.503 | 0.176 | 0.461 | 0.832 | 0.375 | 0.972 | 0.181 |
|  | **v24_1** | **v24_2** | **v24_3** | **v24_4** | **v24_5** | **v24_6** | **v24_7** | **v24_8** | **v24_9** | **v24_10** |
| **v5_1** | 0.301 | 0.794 | 0.404 | 0.792 | 0.397 | 0.670 | 0.860 | 0.669 | 0.544 | 0.447 |
| **v5_2** | 0.781 | 0.640 | 0.435 | 0.845 | 0.426 | 0.718 | 0.798 | 0.715 | 0.488 | 0.397 |
| **v5_3** | 0.908 | 0.973 | 0.058 | 0.969 | 0.531 | 0.847 | 0.686 | 0.836 | 0.405 | 0.329 |
| **v5_4** | 0.778 | 0.842 | 0.443 | 0.575 | 0.434 | 0.718 | 0.813 | 0.715 | 0.507 | 0.416 |
| **v5_5** | 0.951 | 0.889 | 0.681 | 0.895 | 0.155 | 0.992 | 0.573 | 0.973 | 0.328 | 0.266 |
| **v5_6** | 0.498 | 0.555 | 0.239 | 0.557 | 0.238 | 0.043 | 0.889 | 0.454 | 0.763 | 0.637 |
| **v5_7** | 0.494 | 0.549 | 0.243 | 0.551 | 0.242 | 0.441 | 0.679 | 0.451 | 0.789 | 0.664 |
| **v5_8** | 0.705 | 0.767 | 0.393 | 0.766 | 0.386 | 0.647 | 0.895 | 0.164 | 0.581 | 0.480 |
| **v5_9** | 0.283 | 0.324 | 0.119 | 0.327 | 0.121 | 0.243 | 0.584 | 0.258 | 0.734 | 0.945 |
|  | **v24_1** | **v24_2** | **v24_3** | **v24_4** | **v24_5** | **v24_6** | **v24_7** | **v24_8** | **v24_9** | **v24_10** |
| **v13_1** | 0.62 | 0.976 | 0.545 | 0.972 | 0.531 | 0.849 | 0.682 | 0.838 | 0.400 | 0.325 |
| **v13_2** | 0.975 | 0.655 | 0.641 | 0.916 | 0.622 | 0.964 | 0.578 | 0.946 | 0.323 | 0.261 |
| **v13_3** | 0.709 | 0.652 | 0.563 | 0.660 | 0.869 | 0.760 | 0.372 | 0.795 | 0.187 | 0.149 |
| **v13_4** | 0.859 | 0.924 | 0.507 | 0.610 | 0.494 | 0.799 | 0.734 | 0.791 | 0.443 | 0.361 |
| **v13_5** | 0.567 | 0.518 | 0.923 | 0.527 | 0.812 | 0.609 | 0.284 | 0.648 | 0.138 | 0.111 |
| **v13_6** | 0.895 | 0.962 | 0.525 | 0.958 | 0.511 | 0.370 | 0.687 | 0.822 | 0.400 | 0.324 |
| **v13_7** | 0.495 | 0.552 | 0.240 | 0.553 | 0.239 | 0.441 | 0.458 | 0.452 | 0.774 | 0.648 |
| **v13_8** | 0.949 | 0.886 | 0.676 | 0.892 | 0.656 | 0.993 | 0.566 | 0.885 | 0.320 | 0.259 |
| **v13_9** | 0.347 | 0.394 | 0.153 | 0.397 | 0.154 | 0.302 | 0.683 | 0.317 | 0.902 | 0.836 |
| **v13_10** | 0.206 | 0.238 | 0.085 | 0.242 | 0.087 | 0.175 | 0.448 | 0.189 | 0.734 | 0.376 |

**Table 3S.** Wave 1 dataset. AUC on train and test, for glm with different feature sets. Computed for M=10 splits (n_train=789; n_test=336)

| **TRAINING** | | |  | |  | |  | |  |  | |  |  | |  |  | |  |  |  | |
| --- | --- | --- | --- | --- | --- | --- | --- | --- | --- | --- | --- | --- | --- | --- | --- | --- | --- | --- | --- | --- | --- |
| **Model** | | | **X1** | | **X2** | | **X3** | | **X4** | **X5** | | **X6** | **X7** | | **X8** | **X9** | | **X10** | **mean** | **SD** | |
| **R5g** | | | 0.837 | | 0.823 | | 0.836 | | 0.834 | 0.827 | | 0.841 | 0.847 | | 0.831 | 0.833 | | 0.830 | 0.834 | 0.007 | |
| **R5v** | | | 0.822 | | 0.810 | | 0.818 | | 0.822 | 0.817 | | 0.829 | 0.830 | | 0.817 | 0.821 | | 0.819 | 0.821 | 0.006 | |
| **R5p** | | | 0.844 | | 0.822 | | 0.840 | | 0.836 | 0.832 | | 0.844 | 0.850 | | 0.833 | 0.832 | | 0.834 | 0.837 | 0.008 | |
| **R5c** | | | 0.827 | | 0.814 | | 0.823 | | 0.826 | 0.815 | | 0.834 | 0.835 | | 0.814 | 0.816 | | 0.817 | 0.822 | 0.008 | |
| **R6c** | | | 0.837 | | 0.821 | | 0.835 | | 0.833 | 0.827 | | 0.842 | 0.846 | | 0.830 | 0.831 | | 0.829 | 0.833 | 0.007 | |
| **R6b** | | | 0.837 | | 0.821 | | 0.835 | | 0.834 | 0.829 | | 0.842 | 0.847 | | 0.831 | 0.834 | | 0.831 | 0.834 | 0.007 | |
|  | | |  | |  | |  | |  |  | |  |  | |  |  | |  |  |  | |
| **TEST** | | |  | |  | |  | |  |  | |  |  | |  |  | |  |  |  | |
| **Model** | | | **X1** | | **X2** | | **X3** | | **X4** | **X5** | | **X6** | **X7** | | **X8** | **X9** | | **X10** | **mean** | **SD** | |
| **R5g** | | | 0.829 | | 0.858 | | 0.831 | | 0.830 | 0.848 | | 0.821 | 0.806 | | 0.850 | 0.835 | | 0.847 | 0.835 | 0.016 | |
| **R5v** | | | 0.820 | | 0.835 | | 0.822 | | 0.811 | 0.820 | | 0.802 | 0.797 | | 0.835 | 0.816 | | 0.823 | 0.818 | 0.012 | |
| **R5p** | | | 0.821 | | 0.865 | | 0.827 | | 0.833 | 0.845 | | 0.822 | 0.808 | | 0.855 | 0.843 | | 0.846 | 0.836 | 0.017 | |
| **R5c** | | | 0.817 | | 0.838 | | 0.821 | | 0.809 | 0.836 | | 0.803 | 0.795 | | 0.854 | 0.835 | | 0.835 | 0.824 | 0.018 | |
| **R6p** | | | 0.822 | | 0.863 | | 0.829 | | 0.835 | 0.847 | | 0.823 | 0.809 | | 0.855 | 0.842 | | 0.847 | 0.837 | 0.017 | |
| **R6c** | | | 0.829 | | 0.858 | | 0.832 | | 0.830 | 0.849 | | 0.819 | 0.807 | | 0.851 | 0.836 | | 0.847 | 0.836 | 0.016 | |
| **R6b** | | | 0.831 | | 0.860 | | 0.835 | | 0.834 | 0.847 | | 0.822 | 0.809 | | 0.853 | 0.834 | | 0.847 | 0.837 | 0.015 | |
| **FEATURE TEST** | | | | |  | |  | | |  | | |  | | |  | |  | |  |  |
| **Model** | **AGE** | | **GENDER** | | **SatO2** | | **total calcium** | | | **Vol R231** | | | **WAL%_E** | | | **pneumonia_C** | | **GGO-SC_E** | |  |  |
|  |  | |  | |  | |  | | |  | | |  | | |  | |  | |  |  |
| **R5g** | X | | X | | X | | X | | |  | | |  | | |  | | X | |  |  |
| **R5v** | X | | X | | X | | X | | | X | | |  | | |  | |  | |  |  |
| **R5c** | X | | X | | X | | X | | |  | | |  | | | X | |  | |  |  |
| **R5p** | X | | X | | X | | X | | |  | | | X | | |  | |  | |  |  |
| **R6p** | X | | X | | X | | X | | |  | | | X | | |  | | X | |  |  |
| **R6c** | X | | X | | X | | X | | |  | | |  | | | X | | X | |  |  |
| **R6b** | X | | X | | X | | X | | | X | | |  | | |  | | X | |  |  |

**Table 4S.** Delong pairwise comparison of the 10 models built on Var5, Var13, Var24 with R5p (p-values). Computation based on the pROC fast implementation of Delong

|  | **R5p_1** | **R5p_2** | **R5p_3** | **R5p_4** | **R5p_5** | **R5p_6** | **R5p_7** | **R5p_8** | **R5p_9** | **R5p_10** |
| --- | --- | --- | --- | --- | --- | --- | --- | --- | --- | --- |
| **v5_1** | 0.904 | 0.229 | 0.977 | 0.842 | 0.567 | 0.919 | 0.622 | 0.384 | 0.601 | 0.548 |
| **v5_2** | 0.844 | 0.245 | 0.963 | 0.894 | 0.608 | 0.858 | 0.564 | 0.414 | 0.645 | 0.588 |
| **v5_3** | 0.731 | 0.328 | 0.840 | 0.989 | 0.729 | 0.743 | 0.473 | 0.520 | 0.767 | 0.708 |
| **v5_4** | 0.858 | 0.257 | 0.974 | 0.888 | 0.611 | 0.872 | 0.583 | 0.422 | 0.646 | 0.592 |
| **v5_5** | 0.615 | 0.440 | 0.712 | 0.860 | 0.870 | 0.626 | 0.387 | 0.653 | 0.908 | 0.849 |
| **v5_6** | 0.846 | 0.120 | 0.722 | 0.612 | 0.364 | 0.830 | 0.851 | 0.226 | 0.392 | 0.349 |
| **v5_7** | 0.830 | 0.126 | 0.710 | 0.604 | 0.365 | 0.814 | 0.876 | 0.230 | 0.392 | 0.350 |
| **v5_8** | 0.939 | 0.225 | 0.944 | 0.815 | 0.549 | 0.953 | 0.659 | 0.374 | 0.582 | 0.531 |
| **v5_9** | 0.552 | 0.055 | 0.445 | 0.376 | 0.196 | 0.537 | 0.824 | 0.112 | 0.214 | 0.186 |
| **v5_10** | 0.689 | 0.098 | 0.580 | 0.494 | 0.289 | 0.675 | 0.965 | 0.180 | 0.311 | 0.270 |
|  | **R5p_1** | **R5p_2** | **R5p_3** | **R5p_4** | **R5p_5** | **R5p_6** | **R5p_7** | **R5p_8** | **R5p_9** | **R5p_10** |
| **v13_1** | 0.726 | 0.327 | 0.836 | 0.985 | 0.731 | 0.739 | 0.468 | 0.520 | 0.769 | 0.709 |
| **v13_2** | 0.622 | 0.397 | 0.724 | 0.879 | 0.837 | 0.633 | 0.384 | 0.613 | 0.877 | 0.815 |
| **v13_3** | 0.409 | 0.612 | 0.486 | 0.639 | 0.894 | 0.417 | 0.230 | 0.867 | 0.854 | 0.917 |
| **v13_4** | 0.778 | 0.302 | 0.890 | 0.965 | 0.685 | 0.791 | 0.514 | 0.483 | 0.722 | 0.664 |
| **v13_5** | 0.316 | 0.787 | 0.376 | 0.515 | 0.734 | 0.322 | 0.172 | 0.958 | 0.697 | 0.754 |
| **v13_6** | 0.733 | 0.307 | 0.845 | 0.998 | 0.712 | 0.746 | 0.470 | 0.500 | 0.751 | 0.691 |
| **v13_7** | 0.839 | 0.121 | 0.716 | 0.608 | 0.363 | 0.823 | 0.862 | 0.227 | 0.39 | 1 0.348 |
| **v13_8** | 0.608 | 0.433 | 0.706 | 0.857 | 0.869 | 0.619 | 0.379 | 0.648 | 0.908 | 0.847 |
| **v13_9** | 0.647 | 0.072 | 0.532 | 0.450 | 0.245 | 0.631 | 0.934 | 0.144 | 0.266 | 0.234 |
| **v13_10** | 0.422 | 0.039 | 0.333 | 0.283 | 0.141 | 0.410 | 0.657 | 0.080 | 0.154 | 0.134 |
|  | **R5p_1** | **R5p_2** | **R5p_3** | **R5p_4** | **R5p_5** | **R5p_6** | **R5p_7** | **R5p_8** | **R5p_9** | **R5p_10** |
| **v24_1** | 0.643 | 0.379 | 0.746 | 0.902 | 0.813 | 0.654 | 0.400 | 0.590 | 0.853 | 0.791 |
| **v24_2** | 0.704 | 0.341 | 0.812 | 0.963 | 0.753 | 0.716 | 0.450 | 0.539 | 0.791 | 0.731 |
| **v24_3** | 0.343 | 0.701 | 0.410 | 0.559 | 0.798 | 0.350 | 0.186 | 0.964 | 0.759 | 0.819 |
| **v24_4** | 0.703 | 0.351 | 0.810 | 0.959 | 0.760 | 0.715 | 0.452 | 0.548 | 0.798 | 0.739 |
| **v24_5** | 0.338 | 0.747 | 0.402 | 0.544 | 0.771 | 0.344 | 0.186 | 0.998 | 0.734 | 0.792 |
| **v24_6** | 0.585 | 0.410 | 0.684 | 0.844 | 0.868 | 0.595 | 0.352 | 0.635 | 0.909 | 0.846 |
| **v24_7** | 0.956 | 0.165 | 0.834 | 0.713 | 0.451 | 0.941 | 0.749 | 0.293 | 0.482 | 0.434 |
| **v24_8** | 0.588 | 0.457 | 0.683 | 0.834 | 0.897 | 0.599 | 0.365 | 0.676 | 0.935 | 0.875 |
| **v24_9** | 0.629 | 0.071 | 0.517 | 0.437 | 0.238 | 0.614 | 0.913 | 0.140 | 0.259 | 0.227 |
| **v24_10** | 0.522 | 0.057 | 0.423 | 0.359 | 0.191 | 0.508 | 0.777 | 0.112 | 0.208 | 0.182 |

**Table 5S.** Coefficients for glm model R5p

|  | **Estimate Pr(>\|z\|)** | **P value** |
| --- | --- | --- |
| **(Intercept)** | -1.65043 | < 2e-16 *** |
| **Age** | 1.17108 | < 2e-16 *** |
| **Sex** | 0.37703 | 1.78e-05 *** |
| **SatO2** | -0.41790 | 1.61e-06 *** |
| **Total cardiovascular calcium** | 0.24473 | 0.0503 . |
| **WALV%_E** | -0.73584 | 2.04e-14 *** |

Signif. codes: 0 ‘***’ 0.001 ‘**’ 0.01 ‘*’ 0.05 ‘.’
